# Preschool Exposure to Online Games and Internet Gaming Disorder in Adolescents: A Cohort Study

**DOI:** 10.3389/fped.2021.760348

**Published:** 2021-11-23

**Authors:** Hyunsuk Jeong, Hyeon Woo Yim, Seung-Yup Lee, Hae Kook Lee, Marc N. Potenza, Yunmi Shin

**Affiliations:** ^1^Department of Preventive Medicine, College of Medicine, Catholic University of Korea, Seoul, South Korea; ^2^Department of Psychiatry, College of Medicine, Catholic University of Korea, Seoul, South Korea; ^3^Departments of Psychiatry, Neuroscience and Child Study Center, Yale University, New Haven, CT, United States; ^4^Connecticut Council on Problem Gambling, Wethersfield, CT, United States; ^5^Connecticut Mental Health Center, New Haven, CT, United States; ^6^Department of Psychiatry, School of Medicine, Ajou University, Suwon, South Korea

**Keywords:** preschool, online game, internet gaming disorder, adolescents, cohort

## Abstract

**Objectives:** Although considerable evidence has already been collected on the effects of early initiation of drug/alcohol consumption on addictive behaviors in adolescents, little is known about the impact of early exposure to online games during preschool ages on the risk of internet gaming disorder (IGD). We evaluated the effects of exposure to online games before entering elementary school on IGD occurrence during the secondary school years using a community-based longitudinal study.

**Methods:** Data from 1,760 adolescents (seventh grade), who were recruited from the iCURE study and followed for 2 years, were analyzed. A high risk of IGD (HRIGD) was assessed by the Internet Game Use Elicited Symptom Screen, a self-reported questionnaire based on the fifth version of DSM-5 IGD criteria. Early exposure to online gaming was defined as when adolescents played online games during their preschool years. A multivariate generalized-estimating-equation model was applied to examine the independent risk factor of the occurrence of HRIGD during the 2-year follow-up period.

**Results:** As compared with the later-exposure group, those with early exposure to online games showed an ~1.7-fold greater incidence of HRIGD over the 2-year follow-ups after adjusting for potential confounders including baseline IGD scores (adjusted relative risk:1.69; 95%confidence interval:1.08–2.66). Pre-specified sensitivity analyses showed that the results were robust.

**Conclusion:** Exposure to online gaming during the preschool years increases the likelihood of occurrence of HRIGD in adolescence. Restricting exposure to online games during the preschool years should be examined as a way to reduce the risk of IGD in adolescents.

**Clinical Trial Registration:** www.clinicaltrials.gov, identifier: NCT02415322.

## Introduction

Online gaming is a widespread recreational activity, irrespective of culture, age, and gender. However, concerns have been raised regarding whether early exposure to online games may lead to harmful effects on physical and mental health in adolescence (1–3). Among video gaming and various internet activities, online gaming appears to be associated with increased propensities for addiction (4), with much existing research focusing on negative consequences of gaming (5, 6).

Internet gaming disorder (IGD) has been conceptualized based on the theoretical frameworks of substance use disorders (SUDs) (7). These frameworks consider gaming behavior as potentially addictive (8). Early initiation of drug use has been associated with multiple problems later in life, such as negative health, social, and behavioral outcomes and increased likelihoods to develop SUDs (9).

DeWit et al. reported that the likelihood of developing lifetime alcohol dependence in those who start to drink at earlier ages was nearly 10 times that of those who started drinking later on (10). According to results from the New Zealand birth cohort, children who had been introduced to alcohol before the age of 6 years were 2.4 times more likely to report heavy or problem drinking at the age of 15 years than those who did not drink alcohol before the age of 13 years (11). Early age at gambling onset has also been linked to at-risk/problematic gambling during adolescence, particularly with respect to non-strategic forms of gambling involving assume luck without decision making any skill, gamblers cannot influence the outcome of the game (12). Addictive behaviors have been linked to sensation seeking in substance use behavior (6), and a sensation-seeking tendency has also been associated with problematic gaming in adolescents (5).

Although some studies have demonstrated beneficial effects of playing video games on psychological and physical health (13), there is evidence that if used in excess it can become an addictive behavior. Previous researches on video games has focused on the negative effects on gamers. It has been suggested that excessive video gaming is associated with attention problems, poor academic performance, anxiety, depressive symptoms, and deterioration of interpersonal relationships, family conflicts, youth violence or crimes (14). Since adolescence is typically viewed as a life stage where vulnerability to addiction is more pronounced, IGD may lead to particularly serious health problems in adolescents. More specifically, because of cognitive, social, hormonal, and neurobiological immaturities, adolescence is a period of increased risk of experiencing psychological disorders including addictive behaviors (15). Gaming disorder manifests as an impaired control over gaming and an increasing priority over other life interests and daily activities, leading to recurrent gaming despite increasing negative consequences (16, 17).

Although considerable evidence has already been produced on the effects of early initiation of drug or alcohol consumption on addictive behaviors in adolescents, little is known about the impact of early exposure to online games during the preschool ages on the risk of IGD. To fill this gap, we evaluated the relationship between exposure to online games before entering elementary school on the incidence of IGD during the secondary school years in a longitudinal, community-based cohort study.

## Methods

### Study Population

Our study population was derived from the internet user Cohort for Unbiased Recognition of gaming disorder in Early adolescence (iCURE) study, which is a Korean school-based prospective cohort study. A total of 2,319 students in the third, fourth, and seventh grades were enrolled between March 2015 and August 2017. Follow-up assessments were conducted at 12 and 24 months, with follow-up rates of 95% (*n* = 2,206) and 92% (*n* = 2,129), respectively. We included seventh grade adolescents at baseline (*n* = 1,920). Among them, 160 with confirmed high risk of IGD (HRIGD) at baseline were excluded; thus, the study sample included 1,760 normal game user in seventh grade students, who were evaluated with respect to the effects of early exposure to online games on the incidence of HRIGD in the eighth and ninth grades. Written informed consent was acquired from all participants and their parents or legal guardians after they received an explanation of the nature of the principles of research, including confidentiality and the freedom of choice to participate. The pre-registered study protocol has been described in detail elsewhere (18). This analytic study was fully reviewed and approved by the institutional review board of the Catholic University of Korea (MC21EISI0065).

### Measurements

Data collection was performed at participants' schools during school hours during both baseline and follow-up time points. All participants completed questionnaires using self-administered web-based surveys, with a supervising research assistant available to answer questions.

### Incidence of IGD Symptomatology

The incidence of IGD symptomatology was assessed by the Internet Game Use Elicited Symptom Screen (IGUESS). Originally, the IGUESS incorporated DSM-5 IGD diagnostic criteria into a brief self-reported assessment tool, asking questions on experiences regarding nine IGD symptoms during the past 12 months. IGUESS has 9-items. Each item in the IGUESS is rated on a four-point Likert scale (0 = not at all, 1 = occasionally, 2 = frequently, and 3 = always), scores ranged from 0 to 27 points. When comparing the clinician's diagnosis based on the DSM-5 IGD criteria, the sensitivity, specificity, and diagnostic accuracy of the IGUESS were 86.7, 80.0, and 86.8%, respectively, at a cutoff score of 10 points to designate a respondent as HRIGD. Cronbach's alpha provided an index of reliability, and the value of the nine items on the IGUESS was 0.94 (19). IGD symptomatology was assessed at 12 and 24 months. Herein, this scale was deemed reliable, with a Cronbach's alpha of 0.85-0.87.

### Exposure to Online Games

The following question was asked to assess the initial exposure to online games at baseline: “When did you first start playing online games?” Respondents were then asked to select from one of the following: preschool; first, second, third, fourth, fifth, sixth, or seventh grade; or never. Those who responded that they played online games during their preschool years were defined as having “early exposure” and those who started playing online games after entering elementary school were defined as having “later exposure” to online games, respectively.

### Sociodemographic Factors

General characteristics of participants, including gender, family structure, and socioeconomic status (SES), were obtained from baseline data. Family structure was categorized as either intact or non-intact, with an intact family defined when the adolescent was living with both parents and a non-intact family defined as when the adolescent was living with only a mother or father or with neither parent due to divorce, death, or parental separation. SES was assessed using the following question posed to the parents: “Which of the following is your family's level of SES (with options ranging from 1 = *lowest* to 7 = *highest*; scores of 1–4 points were categorized as low to moderate and scores of 5–7 points were categorized as high)?

### Depressive Symptoms

Depressive symptoms were assessed with the Children's Depression Inventory at baseline, which has 27-items, scores ranged from 0 to 54 points. We used the Korean version of the Children's Depression Inventory, which has demonstrated good reliability and validity for the assessment of depressive symptoms (20). A total score of 22 points or more was considered to indicate presence of depressive symptoms. The Cronbach's alpha value was 0.89 in this study.

### Attachment to Parents at Baseline

The Inventory of Parent and Peer Attachment—Revised is a 25-item instrument that employs a five-point Likert scale. This instrument is used to assess adolescents' perceptions of relationships with their parents in terms of the degree of mutual trust, quality of communication, and alienation. Possible scores range from 25 to 125 points, with higher scores indicating a greater degree of attachment to the parents (21). The degree of attachment to parents was assessed at baseline. For descriptive purposes, we used median splits to define higher and lower levels of attachment to parents. The Cronbach's alpha value was 0.94 in this study.

### Openness of Communication With Parents at Baseline

To measure the “openness of communication” between adolescents and their parents, we used subscale of the Parent–Adolescent Communication Inventory. It consists of 20-items and scores range from 0 to 60, with higher scores reflecting a greater degree of openness in parent–adolescent communication (22). The degree of openness of communication between adolescents and their parents was assessed at baseline. For descriptive purposes, we used median splits to define higher and lower levels of openness of communication between parents and adolescents. The Cronbach's alpha value was 0.71 in this study.

### Social Relationship Factor: Perceived Social Support at Baseline

The Social Support Appraisals Scale is a 24-item self-report instrument that assesses perceived social support (23). Items are rated on a five-point Likert scale and higher scores reflecting stronger social support. The degree of social support was assessed at baseline. Although social support was considered a continuous predictor, for descriptive purposes, we utilized median splits to define higher and lower levels of social support. The Cronbach's alpha value was 0.94 in this study.

### Online Gaming Activity at Baseline

Gaming time was assessed by questions that inquired about how many hours participants played online games on average weekdays at baseline. We categorized the average time spent playing online games as <60 min, 60-239 min, or 240 or more minutes per day based on data distribution—the median score was 60 min and the time corresponding to 95% was 240 min. Respondents were asked about the titles of the online games they most frequently had played during the past 12 months and we categorized games as either multiplayer or single-player online games based on the characteristics of their content. Also, in response to “Do you use a PC bang?” those who said “yes” were classified as using PC bangs. For reference, a PC bang (Korean Internet café) is a type of LAN gaming center in South Korea where patrons can play multiplayer computer games for an hourly fee.

### Statistical Analysis

Descriptive statistics were characterized frequencies and numbers or mean ± SDs. The analyses incorporated both 12- and 24-month outcomes into a single model using longitudinal generalized-estimating-equation (GEE) regression methods. The incidence of IGD symptomatology was modeled using an IGUESS score of at least 10 points as the threshold score for HRIGD.

GEE was chosen for the analysis given that factors affecting the relationship are time-dependent measures, and it accounted for correlated data with multiple observations per individual (24). The goal is to make inferences about the population when accounting for the within-subject correlation. GEE tells us that preschool exposure to online game was related to incidence of HRIGD at the two different follow-up points (25). This method provides standard errors adjusted by multiple observations per person using an exchangeable correlation structure, making it possible to consider data from every participant follow-up visit in this analysis (26).

GEE analysis was conducted using a binomial regression model with a logit link function. The working correlation matrix fit autoregressive, exchangeable, and unstructured, respectively, and we finally selected an unstructured correlation matrix for the analysis of correlated data based on the model with the minimum QIC statistic.

In the multivariable GEE model, we adjusted for potential confounding factors including, gender, family structure, SES, depressive symptoms, attachment to parents, openness of communication with parents, and social support as well as baseline IGD scores. Online game–related activities at baseline were not included in the multivariate model as confounders since gaming-related activities were considered to be an outcome of early exposure on online games. We used inverse probability of attrition weighting to address missing data due to participant dropout.

To test the robustness of our findings, we conducted pre-specified sensitivity analyses in terms of the definitions of the study population. First, we included all seventh graders regardless of their baseline IGD scores to evaluate the lifetime cumulative effect of early exposure to online games on IGD without adjusting for baseline IGD scores.

**Figure 1:**
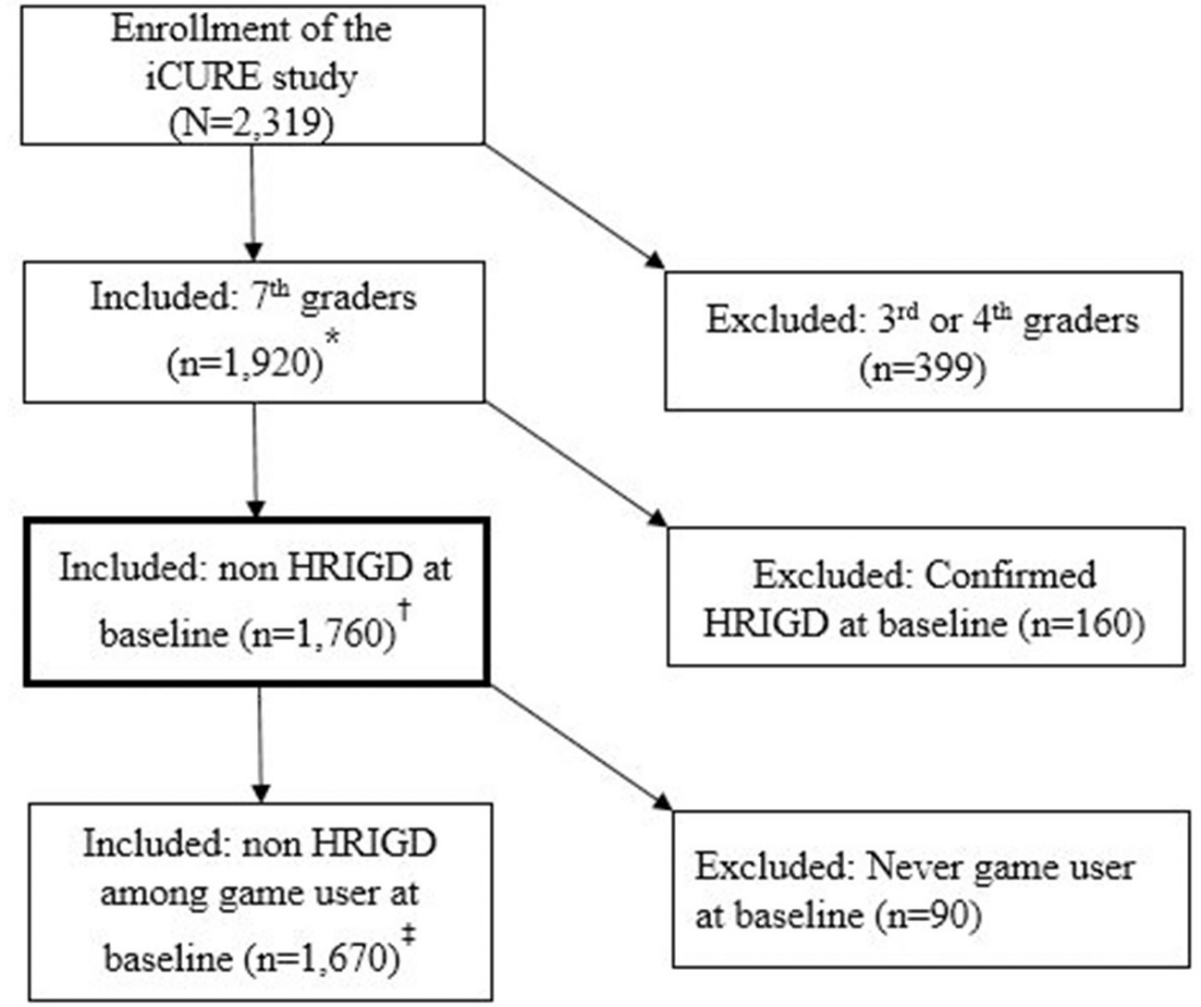
Flow diagram of inclusion and exclusion criteria for the data analysis. *Sensitivity analysis set to evaluate effect of cumulative risk of early exposure to online game on HRIGD. ^†^Main analysis set to evaluate the effect of early exposure to online game on incidence of HRIGD. ^‡^Sensitivity analysis set to evaluate effect of early exposure to online game on incidence of HRIGD among game users at baseline.

Second, we additionally excluded 90 adolescents classified as not using online games at baseline to obtain a conservative estimation of the effects of early exposure to games on the occurrence of IGD symptomatology during the secondary school years. The flow diagram of the data analyses set was depicted in Figure 1. Analyses were performed using SAS software version 9.4 (SAS Institute, Cary, NC, USA). All *p*-values were two-sided.

## Results

Among 1,760 participants, 253 (14.4%) were classified into the group with early exposure to online games and 1,507 (85.6%) were classified into the later-exposure group. The mean age of the participants was 13 years old. There were no between-group differences in gender, family structure, socioeconomic status, depressive symptoms, attachment to parents, openness of communication with parents, and social support (Table 1). However, there were significant differences in online-game–related activities between the two groups in the baseline assessments. As compared with the later-exposure group, adolescents included in the early-exposure group were more likely to use PC bangs, spend more time playing online games on weekdays, and have higher mean baseline IGD assessment scores (all *p* < 0.05) (Table 2).

**Table 1:** Baseline characteristics according to early or later exposure to online games among 1,760 adolescents who have ever played online games.

**Variables**	**Online game exposure**		***P*-value**
	**Early**	**Late**	
	**(*n* = 253, 14.4%)**	**(*n* = 1,507, 85.6%)**	
Age (mean ± SD)	13.0 ± 0.2	13.0 ± 0.2	0.263
Gender			0.279
Boy	135 (53.4)	859 (57.0)	
Girl	118 (46.6)	648 (43.0)	
Family structure			0.727
Intact family	230 (90.9)	1,380 (91.6)	
Non-intact family	23 (9.1)	127 (8.4)	
Socioeconomic status (middle or above)	162 (71.9)	1,048 (69.6)	0.455
Depressive symptoms	12 (4.7)	49 (3.3)	0.230
Lower attachment to parents	133 (52.6)	734 (48.7)	0.255
Lower openness of communication with parents	133 (52.8)	715 (47.5)	0.131
Lower social support	121 (47.8)	734 (48.7)	0.796

*Early exposure was defined as having started to play online games during the preschool years*.

*Later exposure was defined as having started to play online games after entering elementary school*.

**Table 2:** Baseline gaming–related activity according to early or later exposure to online games among 1,760 adolescents who have ever played online games.

**Variables**	**Online game exposure**		***P*-value**
	**Early**	**Late**	
	**(*n* = 253, 14.4%)**	**(*n* = 1,507, 85.6%)**	
PC bang use	61 (24.1)	269 (17.9)	0.018
Game time during weekday (min/day)			0.005
<60	123 (48.6)	749 (48.7)	
60–239	105 (41.5)	685 (45.5)	
≥240	25 (9.9)	73 (4.8)	
Most frequently played type of online game			0.082
None	87 (34.4)	614 (40.7)	
Single-player game	97 (38.3)	563 (37.4)	
Multiple-payer game	69 (27.2)	330 (21.9)	
IGD score (mean ± SD)	3.2 ± 2.7	2.7± 2.6	0.003

*Baseline gaming–related activity was evaluated in the seventh grade (baseline)*.

*IGD scores were evaluated using the IGUESS scale*.

*P-values were calculated by either the chi-squared test or t-test*.

**Figure 2:**
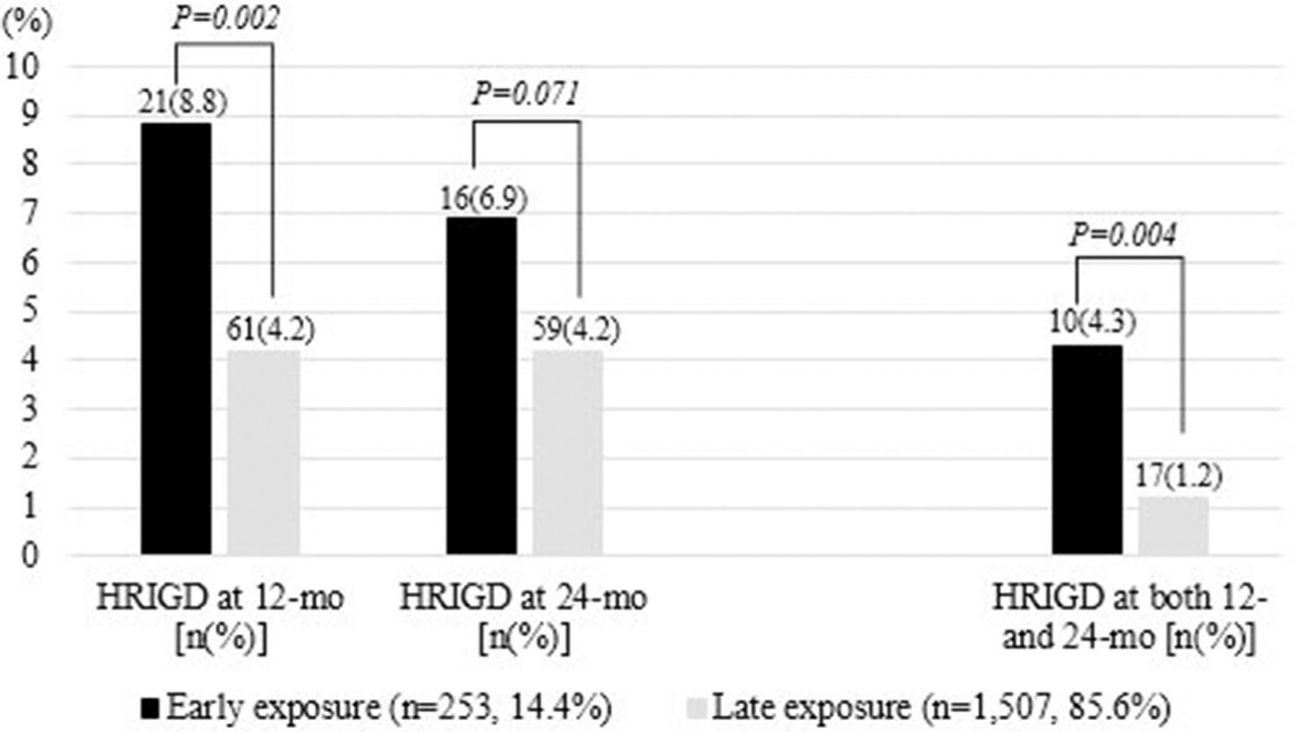
Incidence of HRIGD in the early- and later-exposure groups at 12 and 24 months of follow-up. HRIGD, high risk of internet gaming disorder.

Among 1,760 non-HRIGD participants at baseline, the incidence of HRIGD in the early- and later-exposure groups were 8.8 and 4.2% (*p* = 0.002) at 12 months of follow-up, 6.9 and 4.2% (*p* = 0.071) at 24-months follow-up, and 4.3 and 1.2% (*p* = 0.001) at both 12- and 24-months follow-up (Figure 2).

In the multivariable GEE model, exposure to online games during the preschool years was revealed to be an independent risk factor associated with incidence of HRIGD during secondary school. As compared with in the later-exposure group, those with early exposure to online games showed a 1.69-fold higher incidence of HRIGD after adjusting for sociodemographic, psychological, family, and social relationship factors as well as baseline IGD scores (aRR: 1.69; 95%CI: 1.08–2.66 for model II) (Table 3).

**Table 3:** Univariable and multivariable GEE analyses using binomial models of the relative risks of incidence of HRIGD in the eighth and/or ninth grades among non-HRIGD adolescents in seventh grade (*n* = 1,760/5,280 observations).

	**Model I**	**Model II**
**Online game exposure**	**cRR (95% CI)**	**aRR (95% CI)**
Early (*n* = 253, 14.4%)	1.85 (1.18–2.90)	1.69 (1.08–2.66)
Later (*n* = 1,507, 85.6%)	1	1

*Model I, Crude values*.

*Model II, Adjusted by demographic, psychological, family environment, and social relationship factors, including gender, family structure, socioeconomic status, depressive symptoms, attachment to parents, openness of communication with parents, and social support as well baseline IGD score (seventh grade)*.

*cRR, crude relative risk; aRR, adjusted relative risk; HRIGD, high risk of Internet gaming disorder; CI, confidence interval*.

The first sensitivity analysis included all of the participants to evaluate the cumulative effects of early exposure to online games on the incidence of IGD symptomatology during secondary school. As compared with in the later-exposure group, those with early exposure to online games showed a 2.2-fold increase in HRIGD (95%CI: 1.64–2.97) over the 2-year follow-up period after adjusting for possible confounders (Supplementary Table 1).

A total of 1,670 adolescents classified with current use of online games in the non-HRIGD population at baseline were included in the second sensitivity analysis to evaluate conservative effect estimates of the exposure to games during the preschool years on the incidence of IGD symptomatology. As compared with the later-exposure group, those with early exposure to online games showed an approximately 1.7-fold increase in HRIGD onset(95%CI: 1.07–2.64) over the 2-year follow-up period after adjusting for possible confounders as well as baseline IGD scores (Supplementary Table 2).

## Discussion

Online games have gained popularity since the start of the new millennium and have led to significant growth in the gaming industry (27). South Korea was ranked recently as one of the countries with the greatest percentage of smartphone owners and mobile games were the most popular form of gaming in a global survey (28).

The iCURE cohort was conducted as a baseline survey for seventh-grade students in 2015 and 2016. Most participants were adolescents born between 2002 and 2003. Since online games such as MMORPGs were developing at that time, it is possible that they have been exposed to online game during their preschool years. This population therefore might be a particularly relevant cohort in which to evaluate possible effects of early exposure to online games during the preschool years on the development of IGD symptomatology during adolescence.

In our cross-sectional analysis, no difference in sociodemographic characteristics between non-HRIGD adolescents in the early- and later-exposure groups was found. However, game-related factors differed between the two groups, suggesting possible cumulative effects of game exposure during the preschool years. Since the iCURE study is a school-based cohort, adolescents who had already experienced IGD before the time of cohort enrollment due to early exposure to online games may either had already moved to an alternative school system or refused to participate in the cohort study. Such possibilities may have influenced observed associations at baseline.

In order to estimate the occurrence of IGD risk during the 2-year follow-up period according to early exposure to online games among youth with normal game use, the baseline IGD score was adjusted as a potential confounder. Our 2-year follow-up results suggested that exposure to online games during the preschool years was an independent risk factor for the onset of HRIGD during the secondary school years.

Of 1,760 students classified as non-HRIGD individuals at baseline, there were 253 adolescents who were exposed to online games during their preschool years, and 27 experienced HRIGD during the 2-year follow-up period. The incidence of HRIGD in the early-exposure group was 10.7%. On the other hand, there were 1,507 students who were exposed to online games after entering elementary school, of whom 103 experienced HRIGD during the 2-year follow-up period. The incidence of HRIGD in the later-exposure group was 6.8%. Our results showed that early exposure to online games yields a significant risk ratio of 1.69. The clinical implication is that if early exposure to online games led to an increased HRIGD incidence in adolescents and the early exposure rate was 10% in a group of 10,000 adolescents, then interventions for suppressing exposure to online games during preschool years might be able to prevent approximately 77 non-HRIGD secondary school students from transitioning to an HRIGD status.

Available studies indicate that individuals who begin using the internet at younger ages may exhibit an increased risk for internet addiction (29). Although IGD is considered a behavioral addiction, multiple similarities have been described between addictive gaming and other addictions, such as SUDs and gambling disorder. Excessive game use has been proposed to relate to reward deficiency that may involve reduced dopaminergic activity, similar to SUDs like cocaine-use disorder (30). In a small study, game-playing was found to induce dopamine release similar to that seen with cocaine use (31).

A recent study showed that increased screen-based media consumption was linked to reduced microstructural integrity of brain white-matter tracts, which are important in the early years of brain development, among 5-year-old preschool children (32). Early childhood is a time of rapid brain development; structural connections increase as brain networks become more segregated and specialized (1). These developments are related to a wide range of cognitive developments and are associated with self-regulatory processes. The American Academy of Pediatrics recommends no screen time for children until 18 to 24 months of age and an hour or less of screen time per day for preschool-aged children (33).

We evaluated the cumulative effects of early exposure to online games from the preschool years to the secondary school years and found that the risk score for incident HRIGD was not altered in the sensitivity analysis. Despite the possibility that a selection bias was introduced in this analysis, early exposure to online games was an independent risk factor that increased the risk of IGD symptomatology by 2.2-fold as compared with the later-exposure group.

Given potential debate regarding whether youth without use of online games at baseline carry considerable risk for IGD given absence of exposure, we conducted another sensitivity analysis including only adolescents who played online games at baseline to assess for incident HRIGD. Children who were exposed to online games during their preschool years appeared still significantly at greater risk of HRIGD during their secondary school years. These pre-specified sensitivity analyses supported the robustness of the primary analysis.

There are study limitations. The initiation age of online games was assessed *via* self-report retrospectively and was not independently verified. Among adolescents who reported that they initiated online game-playing during their preschool years, even though the spectrum of game exposure may have varied, early exposure to online games was found to increase the likelihood of HRIGD during adolescence. Psychosocial factors such as depressive symptoms, attachment, and social support were measured in the seventh grade at the time of cohort registration, so they may not accurately reflect the psychosocial characteristics at the time of game exposure. If the psychosocial characteristics such as attachment or depressive symptoms were known at the time of exposure to games, the influence of various psychosocial factors between game exposure and the occurrence of game disorders could have been evaluated.

Since exposure was defined as a broad spectrum, the association between early exposure to online games and the development of HRIGD should be investigated further using finer assessments.

We used the cutoff value on a median split of psychosocial measurements, including attachment to parents, openness of communication with parents, and perceived social support. Compared with the original continuous measure, an artificially categorized variable can be less precise because it does not allow the researcher to discriminate between differently scoring members in the same group.

## Conclusion

Early exposure to online games continues to increase the likelihood of incident HRIGD during the secondary school years among youth with normal game-playing in the seventh grade. Restricting exposure to online games during the preschool years should be investigated further as a possible way to reduce the risk of IGD in adolescents.

As a strategy to reduce the risk of IGD in adolescents, it is necessary to provide information to children and parents about the possibility that early exposure to online games can increase the risk of IGD. Because parent–child communication and parental monitoring regarded as one central parental skill to focus on prevention of IGD, interventions or counseling that focus on parental involvement can yield behavioral change in children.

## Data Availability Statement

The raw data supporting the conclusions of this article will be made available by the authors, without undue reservation.

## Ethics Statement

Written informed consent was acquired from all participants and their parents or legal guardians after they received an explanation of the nature of the principles of research, including confidentiality and the freedom of choice to participate. This study was fully reviewed and approved by the Institutional Review Board of the Catholic University of Korea (MC21EISI0065). Written informed consent to participate in this study was provided by the participants' legal guardian/next of kin.

## Author Contributions

HJ conceptualized and designed the study, designed the data collection instruments, developed data analysis plan, carried out the initial analyses, interpreted the data for the work, drafted the initial manuscript, and reviewed and revised the manuscript. HY conceptualized and designed the study, designed the data collection instruments, interpreted the data for the work, guided and supervised the writing of the manuscript, and reviewed and revised the manuscript. S-YL and HL designed the data collection instruments, coordinated and supervised data collection, and reviewed and revised the manuscript. MP and YS interpreted the data for the work and reviewed critically the manuscript for important intellectual content, and revised the manuscript. All authors approved the final manuscript as submitted and agree to be accountable for all aspects of the work.

## Funding

This study was supported by a grant from the Korean Mental Health Technology R&D Project, Ministry of Health and Welfare, Republic of Korea (HL19C0012). The Ministry of Health and Welfare had no further role in study design; in the collection, analysis and interpretation of data; in the writing of the report; or in the decision to submit the paper for publication. MP has received support from the Connecticut Council on Problem Gambling.

## Conflict of Interest

MP has consulted for and advised Opiant Pharmaceuticals, Idorsia Pharmaceuticals, AXA, Game Day Data, and the Addiction Policy Forum; has been involved in a patent application with Yale University and Novartis; has received research support from the Mohegan Sun Casino, the Connecticut Council on Problem Gambling, and the National Center for Responsible Gaming; has participated in surveys, mailings or telephone consultations related to drug addiction, impulse control disorders or other health topics; and has consulted for law offices and gambling entities on issues related to impulse control or addictive disorders. The remaining authors declare that the research was conducted in the absence of any commercial or financial relationships that could be construed as a potential conflict of interest.

## Publisher's Note

All claims expressed in this article are solely those of the authors and do not necessarily represent those of their affiliated organizations, or those of the publisher, the editors and the reviewers. Any product that may be evaluated in this article, or claim that may be made by its manufacturer, is not guaranteed or endorsed by the publisher.

## Abbreviations

IGD: Internet gaming disorder
HRIGD: High risk of Internet gaming disorder
iCURE: Internet User Cohort for Unbiased Recognition of Gaming Disorder in Early Adolescence
IGUESS: Internet Game Use-Elicited Symptom Screen
DSM−5: The fifth version of the Diagnostic and Statistical Manual of Mental Disorders
ICD-11: The 11th revision of the International Classification of Diseases
SES: Socioeconomic status
GEE: Generalized estimating equation.

## References

[1] Mills KL Goddings AL Herting MM Meuwese R Blakemore SJ Crone EA. Structural brain development between childhood and adulthood: convergence across four longitudinal samples. Neuroimage. (2016) 141:273-81. 10.1016/j.neuroimage.2016.07.044 27453157 PMC5035135

[2] Wang JL Sheng JR Wang HZ. The association between mobile game addiction and depression, social anxiety, and loneliness. Front Public Health. (2019) 7:247. 10.3389/fpubh.2019.00247 31552213 PMC6743417

[3] Jeong H Yim HW Lee SY Lee HK Potenza MN Lee H. Factors associated with severity, incidence or persistence of internet gaming disorder in children and adolescents: a 2-year longitudinal study. Addiction. (2020) 116:1828-38. 10.1111/add.15366 33283397

[4] Lemmens JS Hendriks SJF. Addictive online games: examining the relationship between game genres and Internet gaming disorder. Cyberpsychol Behav Soc Netw. (2016) 19:270-6. 10.1089/cyber.2015.0415 26959285

[5] Hu J Zhen S Yu C Zhang Q Zhang W. Sensation seeking and online gaming addiction in adolescents: a moderated mediation model of positive affective associations and impulsivity. Front Psychol. (2017) 8:699. 10.3389/fpsyg.2017.00699 28529494 PMC5418345

[6] Jamt REG Gjerde H Furuhaugen H Romeo G Vindenes V Ramaekers JG. Associations between psychoactive substance use and sensation seeking behavior among drivers in Norway. BMC Public Health. (2020) 20:23. 10.1186/s12889-019-8087-0 31914964 PMC6950984

[7] Petry NM O'Brien CP. Internet gaming disorder and the DSM-5. Addiction. (2013) 108:1186-7. 10.1111/add.12162 23668389

[8] Griffiths MD Kuss DJ Lopez-Fernandez O Pontes HM. Problematic gaming exists and is an example of disordered gaming. J Behav Addict. (2017) 6:296-301. 10.1556/2006.6.2017.037 28816501 PMC5700713

[9] Trujillo CA Obando D Trujillo A. An examination of the association between early initiation of substance use and interrelated multilevel risk and protective factors among adolescents. PLoS ONE. (2019) 14:e0225384. 10.1371/journal.pone.0225384 31825955 PMC6905657

[10] DeWit DJ Adlaf EM Offord DR Ogborne AC. Age at first alcohol use: a risk factor for the development of alcohol disorders. Am J Psychiatry. (2000) 157:745-50. 10.1176/appi.ajp.157.5.745 10784467

[11] Fergusson DM Lynskey MT Horwood LJ. Childhood exposure to alcohol and adolescent drinking patterns. Addiction. (1994) 89:1007-16. 10.1111/j.1360-0443.1994.tb03360.x 7950847

[12] Rahman AS Pilver CE Desai RA Steinberg MA Rugle L Krishnan-Sarin S. The relationship between age of gambling onset and adolescent problematic gambling severity. J Psychiatr Res. (2012) 46:675-83. 10.1016/j.jpsychires.2012.02.007 22410208 PMC3334397

[13] Primack BA Carroll MV McNamara M Klem ML King B Rich M. Role of video games in improving health-related outcomes: a systematic review. Am J Prev Med. (2012) 42:630-8. 10.1016/j.amepre.2012.02.023 22608382 PMC3391574

[14] Paulus FW Ohmann S von Gontard A Popow C. Internet gaming disorder in children and adolescents: a systematic review. Dev Med Child Neurol. (2018) 60:645-59. 10.1111/dmcn.13754 29633243

[15] Jeong H Lee H Kwon Y Yim H Lee S. Gaming disorder and bidirectional relationships with aggression and impulsivity. Curr Opin Behav Sci. (2020) 31:69-75 10.1016/j.cobeha.2019.12.003

[16] American Psychiatric Association. Diagnostic and Statistical Manual of Mental Disorders –Text Revision. 5th ed. Washington, DC: American Psychiatric Association (2013).

[17] Reed GM First MB Kogan CS Hyman SE Gureje O Gaebel W. Innovations and changes in the ICD-11 classification of mental, behavioural and neurodevelopmental disorders. World Psychiatry. (2019) 18:3-19. 10.1002/wps.20611 30600616 PMC6313247

[18] Jeong H Yim HW Jo SJ Lee SY Kim E Son HJ. Study protocol of the internet user Cohort for Unbiased Recognition of gaming disorder in Early adolescence (iCURE), Korea, 2015-2019. BMJ Open. (2017) 7:e018350. 10.1136/bmjopen-2017-018350 28982839 PMC5640066

[19] Jo SJ Yim HW Lee HK Lee HC Choi JS Baek KY. The internet game use-elicited symptom screen proved to be a valid tool for adolescents aged 10-19 years. Acta paediatrica. (2017) 107:511-6. 10.1111/apa.14087 28940637

[20] Cho S Choi J. Development of state - trait anxiety scale for Korean children. Med J Seoul Natl Univ. (1989) 14:150-7.

[21] Armsden GC Greenberg MT. The inventory of parent and peer attachment: individual differences and their relationship to psychological well-being in adolescence. J Youth Adolesc. (1987) 16:427-54. 10.1007/BF02202939 24277469

[22] Bienvenu MJ. A Parent-Adolescent Communicaiton Inventory. Saluda, NC: Family Life publictions (1969).

[23] Dubow EF Tisak J. The relation between stressful life events and adjustment in elementary school children: the role of social support and social problem-solving skills. Child Dev. (1989) 60:1412-23. 10.2307/1130931 2612250

[24] Zeger SL Liang KY. Longitudinal data analysis for discrete and continuous outcomes. Biometrics. (1986) 42:121-30. 10.2307/2531248 3719049

[25] Hoogendoorn WE Bongers PM de Vet HC Twisk JW van Mechelen W Bouter LM. Comparison of two different approaches for the analysis of data from a prospective cohort study: an application to work related risk factors for low back pain. Occup Environ Med. (2002) 59:459-65. 10.1136/oem.59.7.459 12107294 PMC1740320

[26] Hanley JA Negassa A Edwardes MD Forrester JE. Statistical analysis of correlated data using generalized estimating equations: an orientation. Am J Epidemiol. (2003) 157:364-75. 10.1093/aje/kwf215 12578807

[27] Kuss DJ Louws J Wiers RW. Online gaming addiction? Motives predict addictive play behavior in massively multiplayer online role-playing games. Cyberpsychol Behav Soc Netw. (2012) 15:480-5. 10.1089/cyber.2012.0034 22974351

[28] Pew Research Center. Smartphone Ownership Is Growing Rapidly Around the World, but Not Always Equally. (2019). Available online at: https://wwwpewresearchorg/global/2019/02/05/smartphone-ownership-is-growing-rapidly-around-the-world-but-not-always-equally/ (accessed July 20, 2021).

[29] Koo HJ Kwon JH. Risk and protective factors of internet addiction: a meta-analysis of empirical studies in Korea. Yonsei Med J. (2014) 55:1691-711. 10.3349/ymj.2014.55.6.1691 25323910 PMC4205713

[30] Fauth-Bühler M Mann K. Neurobiological correlates of internet gaming disorder: similarities to pathological gambling. Addict Behav. (2017) 64:349-56. 10.1016/j.addbeh.2015.11.004 26621112

[31] Weinstein N Przybylski AK Murayama K. A prospective study of the motivational and health dynamics of Internet Gaming Disorder. PeerJ. (2017) 5:e3838. 10.7717/peerj.3838 28975056 PMC5624294

[32] Hutton JS Dudley J Horowitz-Kraus T DeWitt T Holland SK. Associations between screen-based media use and brain white matter integrity in preschool-aged children. JAMA Pediatr. (2020) 174:e193869. 10.1001/jamapediatrics.2019.3869 31682712 PMC6830442

[33] Media and young minds. Pediatrics. (2016) 138:e20162591. 10.1542/peds.2016-2591 27940793

