# Supplementary material for: Preschool Exposure to Online Games and Internet Gaming Disorder in Adolescents: A Cohort Study
Source: Front Pediatr. 2021 Nov 23;9:760348. doi: 10.3389/fped.2021.760348 (PMC8650316; doi:10.3389/fped.2021.760348)
Supplement: Supplementary file 1 [file Data_Sheet_1.docx]

Supplementary Table 1. Sensitivity analyses of univariable and multivariable GEE analyses using binomial and Poisson models of the cumulative relative risks of HRIGD in the seventh, eighth, and/or ninth grades among all participants (n = 1,920/5,760 observations)

|  | Model I | Model II | Model III |
| --- | --- | --- | --- |
| Online game exposure | cRR (95% CI) | aRR (95% CI) | aRR (95% CI) |
| Early (n = 300, 15.6%) | 2.48 (1.72–3.57) | 2.52 (1.74–3.64) | 2.21 (1.64–2.97) |
| Later (n = 1,620, 84.4%) | 1 | 1 | 1 |

Model I: Crude values

Model II: Adjusted by demographic factors, including gender, family structure, socioeconomic status, and baseline IGD score

Model III: Adjusted by demographic, psychological, family environment, and social relationship factors, including gender, family structure, socioeconomic status, depressive symptoms, attachment to parents, openness of communication with parents, and social support as well baseline IGD score (seventh grade)

cRR: crude relative risk; aRR: adjusted relative risk; HRIGD: high risk of Internet gaming disorder; CI: confidence interval

Supplementary Table 2. Sensitivity analyses of univariable and multivariable GEE analyses using binomial and Poisson models of the relative risks of HRIGD onset in the eighth and/or ninth grades among non-HRIGD and game users in the seventh grade (n = 1,670/5,010 observations)

|  | Model I | Model II | Model III |
| --- | --- | --- | --- |
| Online game exposure | cRR (95% CI) | aRR (95% CI) | aRR (95% CI) |
| Early (n = 253, 15.2%) | 1.75 (1.12–2.75) | 1.69 (1.08–2.65) | 1.68 (1.07–2.64) |
| Later (n = 1,417, 84.8%) | 1 | 1 | 1 |

Model I: Crude values

Model II: Adjusted by demographic factors, including gender, family structure, socioeconomic status, and baseline IGD score

Model III: Adjusted by demographic, psychological, family environment, and social relationship factors, including gender, family structure, socioeconomic status, depressive symptoms, attachment to parents, openness of communication with parents, and social support as well baseline IGD score (seventh grade)

cRR: crude relative risk; aRR: adjusted relative risk; HRIGD: high risk of Internet gaming disorder; CI: confidence interval
